# Pullulan–Apple Fiber Biocomposite Films: Optical, Mechanical, Barrier, Antioxidant and Antibacterial Properties

**DOI:** 10.3390/polym13060870

**Published:** 2021-03-11

**Authors:** Ângelo Luís, Ana Ramos, Fernanda Domingues

**Affiliations:** 1 Health Sciences Research Centre (CICS-UBI), University of Beira Interior, Avenida Infante D. Henrique, 6200-506 Covilhã, Portugal; fdomingues@ubi.pt; 2 Pharmaco-Toxicology Laboratory, UBIMedical, University of Beira Interior, Estrada Municipal 506, 6200-284 Covilhã, Portugal; 3 Chemistry Department, Sciences Faculty, University da Beira Interior, Rua Marquês d’Ávila e Bolama, 6201-001 Covilhã, Portugal; ammr@ubi.pt; 4 Fiber Materials and Environmental Technologies Research Unit (FibEnTech), University of Beira Interior, Rua Marquês d’Ávila e Bolama, 6201-001 Covilhã, Portugal

**Keywords:** pullulan, apple fiber, biocomposites, films, antioxidant activity, antibacterial properties

## Abstract

More than 150 million tons of synthetic plastics are produced worldwide from petrochemical-based materials, many of these plastics being used to produce single-use consumer products like food packaging. The main goal of this work was to research the production and characterization of pullulan–apple fiber biocomposite films as a new food packaging material. The optical, mechanical, and barrier properties of the developed biocomposite films were evaluated. Furthermore, the antioxidant and antibacterial activities of the biocomposite films were additionally studied. The results show that the Tensile Index and Elastic Modulus of the pullulan–apple fiber films were significantly higher (*p*-value < 0.05) when compared to the pullulan films. Regarding the water vapor permeability, no significant differences (*p*-value < 0.05) were observed in water vapor transmission rate (WVTR) when the apple fiber was incorporated into the biocomposite films. A significant increase (*p*-value < 0.05) of water contact angle in both sides of the films was observed when the apple fiber was incorporated into pullulan, indicating an increase in the hydrophobicity of the developed biocomposite films. It is worth noting the hydrophobicity of the (rough) upper side of the pullulan–apple fiber films, which present a water contact angle of 109.75°. It was possible to verify the microbial growth inhibition around the pullulan–apple fiber films for all the tested bacteria.

## 1. Introduction

Currently, more than 150 million tons of synthetic plastics are produced worldwide from petrochemical-based materials, such as polyolefins and polyesters. Many of these plastics are used to produce single-use consumer products like food packaging [1]. This causes a serious environmental problem, as most of those plastics do not degrade when disposed of in the environment at the end of their life cycle, being recalcitrant to environmental attack [1]. Several studies have focused on finding alternatives for conventional petroleum-derived plastics using biopolymers or low-cost biomass with comparable properties [2]. Biopolymers from agricultural resources, such as starch, cellulose, proteins, and pectin [3], which could be degraded in a few months, can be applied to the sustainable production of packaging materials since they are biodegradable, non-toxic, and recyclable [4].

Contamination of processed foods by foodborne pathogens is an important safety issue for consumers and food processors. To address this question, food-compatible, plant-derived antimicrobials could reduce surface contamination of food products [5]. Antimicrobial edible films produced from plant-derived foods could offer an additional level of protection against microbial spoilage of food, as well as meet the increasing consumer demands for safe and natural foods [6]. The incorporation of natural antioxidants obtained from fruit and plant extracts to enhance the shelf-life of foods has also become a widespread strategy [7].

Despite not being an exceptional dietary fiber source, apples may provide several health benefits related to the synergy of their fiber fractions with other nutrients [8]. Dietary fibers found in apples are of superior quality than those obtained from cereals since they present higher solubility and concentration in additional health-promoting bioactive compounds [8].

Several studies have depicted the application of apple by-products as new raw materials for the development of biodegradable materials such as edible films, which also have antioxidant activity due to the polyphenols present in apples [4]. However, in earlier studies [4,8], films made solely by apple by-products showed a weak film-forming capacity, making their employment unfeasible in food packaging production [4,8]. Due to this, other biopolymers are required to be mixed with apple-derived products to enhance their capability to form films [4].

Pullulan is a water-soluble extracellular polysaccharide produced by *Aureobasidium pullulans* in cultures of sugar and starch [9]. The linear polymer mostly consists of maltotriose units connected to each other by α-(1,6) glycosidic bonds [10]. This single linkage pattern gives pullulan distinct physical properties to form transparent, strong, low-permeability, and water-soluble films [11]. Despite the advantageous properties of pullulan, its use in food applications is frequently hindered due to its high cost. Therefore, pullulan is frequently blended with other biopolymers to reduce the cost, as well as to improve the material properties [11].

The main goal of this work was, therefore, to research the production and characterization of pullulan–apple fiber biocomposite films as a new food packaging material. The optical, mechanical, barrier, antioxidant, and antibacterial properties of the biocomposite films developed were further evaluated.

## 2. Materials and Methods

### 2.1. Reagents

Pullulan (CAS Number: 9057-02-7), with a molar mass of 574.57 g/mol ((C_23_H_42_O_16_)_n_), was supplied by TCI Europe (Zwijndrecht, Belgium). Apple fiber, with a mean particle size of 150 µm, was obtained from My Protein (a THG company) (Voyager House, Manchester; UK). Glycerol (anhydrous) (CAS Number: 56-81-5) was purchased from Merck (Darmstadt, Germany).

### 2.2. Biocomposite Films Preparation

Initially, 2 g of pullulan (2%, *w*/*v*) were mixed with 1 g of apple fiber (1%, *w*/*v*) at room temperature in 100 mL of distilled water. This mixture was stirred using a magnetic stirrer (Heidolph, Schwabach, Germany) (250 rpm) for 15 min. Then, 20% glycerol (*w*/*w*, relative to the biopolymers) was included as a plasticizer in the polymers mixture and stirred at room temperature for 5 min, and at 50 °C for another 30 min. Finally, this mixture was homogenized for 5 min at 10,000 rpm using a disperser IKA T25 Digital Ultra-Turrax (Staufen, Germany). The biocomposite films were obtained by casting 16 mL of the polymer mixture in polystyrene Petri dishes (9 cm of diameter), which were subsequently dried for around 3 h at 60 °C in a forced-air laboratory oven. Pullulan films not including apple fiber were also produced, to be used as a control. Finally, the dried films were detached from the Petri dishes and kept under controlled relative humidity (RH) (50 ± 5%) and temperature (23 ± 2 °C) [1,2,4,12,13].

### 2.3. Surface Morphology of the Films

The surface morphology of the biocomposite films was observed by optical microscopy using a Nikon Labophot-2 microscope (Nikon, Tokyo, Japan) equipped with a Leica MC190 HD camera and (Leica, Wetzlar, Germany) controlled by the LAS v4.13 software (https://imillermicroscopes.com/pages/software-download accessed on 31 January 2021).

### 2.4. Infrared Spectra

A Nicolet iS10 smart iTRBasic Thermo Fisher Scientific (Waltham, MA, USA) was used to obtain the Fourier-Transform Infrared Spectroscopy (FTIR) spectra of the biocomposite films between 600 and 4000 cm^−1^, acquiring 120 scans with 4 cm^−1^ of resolution [12].

### 2.5. Thermal Analysis

A calorimeter Netzsch DSC 204 (GWP, Munich, Germany) was used to obtain the Differential Scanning Calorimetry (DSC) thermograms of the biocomposite films, functioning with the following conditions: an inert atmosphere, a heating rate of 5 °C/min, and a temperature ranging from 25 to 500 °C. Before the study, samples of the films were maintained at 105 °C for 24 h to totally evaporate the water, also being obtained the respective baselines [12].

### 2.6. Grammage, Thickness, Mechanical, and Optical Properties

The biocomposite films’ grammage was determined by the quotient between their mass and area (g/m^2^), according to the ISO 536:1995. An Adamel Lhomargy Model MI 20 micrometer (TMI, Veenendaal, Netherlands) was used to measure the thickness (µm) of the films, considering different random assessments, following the ISO 534:2011 [12,13].

The mechanical properties of the biocomposite films (Peak Elongation (%), Tensile Index (N m/g), Tensile Strength (N/m), and Elastic Modulus (MPa)) were determined using a Thwing-Albert Instrument Co. (West Berlin, NJ, USA) tensile tester, adjusting the crosshead at 10 mm/min, and the initial grip at 50 mm, following the ISO 1924/1 [12,13].

The optical properties of the biocomposite films (color coordinates and transparency) were measured using a Color Touch 2 spectrophotometer (Technidyne, New Albany, MS, USA). The determinations were achieved considering several arbitrary positions of the films using the illuminant D65 (daylight with a UV component) and an observation angle of 10°. Color coordinates L*, a*, and b* (lightness; redness/±red-green; yellowness/±yellow-blue) were obtained [12,13].

### 2.7. Barrier Properties

#### 2.7.1. Oil Permeability

Firstly, 5 mL of edible vegetal oil (obtained from sunflower seeds and composed of 70% linoleic acid, 10% linolenic acid, and 10% oleic acid) (Vitaquell, Hamburg, Germany) were put into test tubes, which were then sealed with the biocomposite films. The tubes were turned upside down on the surface of a filter paper previously dried at 105 °C for 24 h and weighed. The oil permeability (OP) (g mm/m^2^ day) was calculated with the weight difference of the filter paper, the thickness of the biocomposite films, the effective contact area, and the storage period (24 h) according to the following equation:

(1) $$OP=\frac{\Delta W\times e}{A\times T},$$

where *ΔW* is the weight difference of the filter paper (g), *e* corresponds to the thickness of the film (mm), *A* is the contact area (m^2^), and *T* is the storage period (days) [14].

#### 2.7.2. Water Vapor Permeability

The water vapor permeability (WVP) (g/Pa day m) and the water vapor transmission rate (WVTR) (g/m^2^ day) were evaluated following the ASTM E96-00 standard procedure. For that, equilibrated test cups containing a desiccant (15 g of anhydrous CaCl_2_ (Sigma-Aldrich, MO, USA), previously dried at 105 °C) were sealed with the biocomposite films. Then, the cups were put in a container at 23 ± 2 °C and 50 ± 5% RH, the weight differences being checked every 2 h for 48 h. The slope of a linear regression of the weight increase versus time was used to determine the gradient [15]. The WVTR and WVP were calculated using Equations (2) and (3):

(2) $$WVTR=\frac{\frac{\Delta m}{\Delta T}}{A},$$

where *∆m* is the weight change of the test cups (g), *T* is the test time (h), and *A* is the test area (m^2^).

(3) $$WVP=\frac{WVTR}{\Delta p}=\frac{WVTR}{p\times \left(RH_{1}-RH_{2}\right)}\times e,$$

where *p* is the water vapor pressure at 23 °C (Pa), *RH_1_* is the RH of the container (50%), *RH_2_* is the RH inside the cups (0%), and *e* is the thickness (m) of the biocomposite films.

### 2.8. Contact Angle and Surface Free Energy

The values of the contact angles of the biocomposite films were measured by the sessile drop method using an OCAH 200 model from DataPhysics Instruments (Filderstadt, Germany), which allowed for image acquisition and data analysis at the same time [12,13]. The surface free energy (total, dispersive, and polar components) of the biocomposite films was calculated by determining the contact angles using three reference liquids (distilled water, diiodomethane, and ethylene glycol) [16]. The surface free energy components of these liquids were obtained directly from the software [17]. Contact angles were taken from six measurements for each liquid and each sample, considering random positions of the biocomposite films, the Owens-Wendt approach being employed in the obtention of the surface free energies of the biocomposite films [18].

### 2.9. Antioxidant Activity

#### 2.9.1. DPPH Free Radical Scavenging Assay

For this assay, 2.9 mL of a DPPH (2,2-diphenyl-1-picrylhydrazyl) (Sigma-Aldrich, St. Louis, MO, USA) solution (0.1 mM in methanol) were mixed with 3 disks of the biocomposite films (6 mm in diameter). Then, the absorbances of these mixtures were measured every 30 min for 5 h at 517 nm against methanol (Sigma-Aldrich, St. Louis, MO, USA) as a blank. The mixture of 2.9 mL of the DPPH solution with 100 µL of methanol was used as the control sample. The antioxidant activity of the biocomposite films was calculated using the following equation [13,19]:

(4) $$\%Inhibition=\frac{A_{control}-A_{sample}}{A_{control}}\times 100,$$

where *A_control_* is the absorbance of the control, and *A_sample_* is the absorbance of the samples (films).

#### 2.9.2. β-Carotene Bleaching Test

Firstly, 50 µL of a β-carotene solution (20 mg/mL in chloroform (Sigma-Aldrich, MO, USA)) were mixed with linoleic acid (40 µL), Tween 40 (400 µL), and chloroform (1 mL), the chloroform being evaporated under vacuum. Then, oxygenated distilled water (100 mL) was added to the residue, forming an emulsion. Subsequently, 5 mL of the emulsion were mixed with 3 disks of the biocomposite films (6 mm in diameter) in test tubes. Finally, the tubes were placed in a 50 °C environment for 1 h. The absorbances of these samples were measured at 470 nm against a blank containing an emulsion prepared without the β-carotene. The mixture of 5 mL of the emulsion with 300 µL of methanol was used as the control sample. The antioxidant activity of the biocomposite films was calculated as the percentage of inhibition of β-carotene oxidation using the following equation [13,19]:

(5) $$\%Inhibition=\frac{A_{sample}^{t=1h}-A_{control}^{t=1h}}{A_{control}^{t=0h}-A_{control}^{t=1h}}\times 100,$$

where *A^t=1h^* is the absorbance of the samples (films) or the control at the final time of reaction, and *A^t=0h^* is the absorbance of the control at the initial time of reaction.

### 2.10. Antibacterial Properties

The antibacterial activity of the biocomposite films against seven foodborne pathogens (*Staphylococcus aureus* ATCC 25923, *Listeria monocytogenes* LMG 16779, *Enterococcus faecalis* ATCC 29212, *Bacillus cereus* ATCC 11778, *Salmonella* Typhimurium ATCC 13311, *Escherichia coli* ATCC 25922, and *Pseudomonas aeruginosa* ATCC 27853) was evaluated by solid assay. Stock cultures of the bacterial species were maintained at −80 °C with 20% (*v*/*v*) glycerol. All the bacterial species were cultivated in brain–heart infusion agar (BHI) for 24 h before the antibacterial tests [19]. For the solid assay, several microbial colonies were suspended in a sterile saline solution (NaCl (Sigma-Aldrich, MO, USA); 0.85%; *w*/*v*), adjusting the suspension of the inoculums to 0.5 McFarland (≈1.5 × 10^8^ colony-forming units (CFU)/mL). Disks of the biocomposite films (6 mm in diameter) were cut under asepsis. Then, the prepared disks were placed on the inoculated Müeller-Hinton agar (MHA) or BHI plates. Lastly, the plates were incubated for 18 h at 37 °C. After the incubation period, they were visually examined for inhibition zones and their diameters were measured with a pachymeter [12,13]. Additionally, the plates were observed by optical microscopy, as described above, to verify the microbial growth inhibition and the integrity of the films after the incubation period. The results were achieved by three independent assays.

### 2.11. Biodegradability

To evaluate the biodegradability of the biocomposite films, a soil burial degradation test was performed. Small strips of the biocomposite films (2 × 7 cm) were interred in organic soil at a depth of 10 cm and maintained at 50 ± 5% RH and 23 ± 2 °C for 10 days. Afterwards, the strips were collected, cleaned with distilled water, and dried in an oven (50 °C) for 24 h. The weight loss (*WL*) was determined using the following equation [15,20]:

(6) $$WL\left(\%\right)=\frac{W_{sample}^{initial}-W_{sample}^{final}}{W_{sample}^{initial}}\times 100,$$

where *W* corresponds to the weight of the samples before and after the soil burial degradation test.

### 2.12. Statistical Analysis

Overall, the results are shown as the mean ± standard deviation (SD). The IBM SPSS Statistics v25 software (https://www.ibm.com/analytics/spss-statistics-software accessed on 7 December 2020) was used to analyze the raw values, employing the Student’s T-test (assuming the normal distribution of the continuous variables). Differences among means are considered to be significant if the *p*-value is <0.05 (a confidence level of 95%).

## 3. Results and Discussion

### 3.1. Appearance and Surface Morphology of the Films

**Figure 1:**
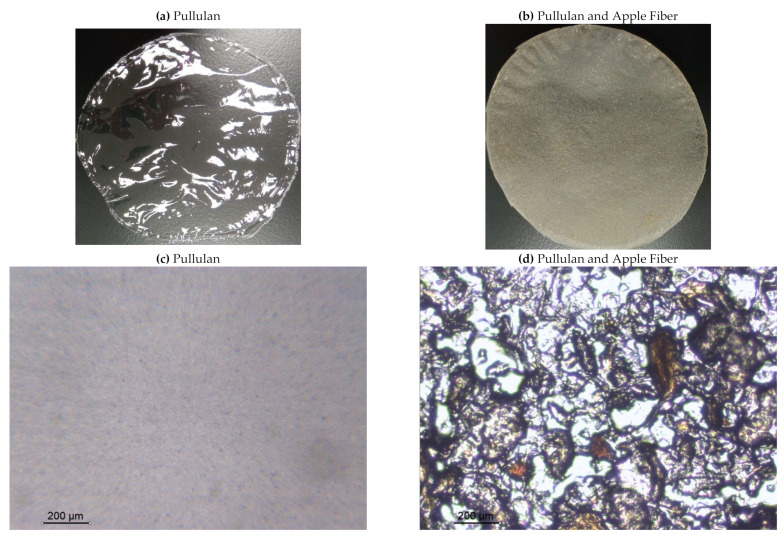
Appearance of the pullulan film (**a**), pullulan and apple fiber film (**b**), and surface morphology (optical microscopy, magnification: 400×) of the pullulan film (**c**), pullulan and apple fiber film (**d**).

The pullulan films appeared to be colorless, transparent, and homogenous (Figure 1a), presenting a smooth surface with compact structural integrity (Figure 1c). On the other hand, the pullulan–apple fiber films were yellowish, although transparent (Figure 1b), with the apple fiber uniformly dispersed in the pullulan matrix. In addition, these films had a rough surface on the upper side of the film (Figure 1d). In both types of the developed films, there were no pinholes formation, which will particularly affect their barrier properties.

### 3.2. FTIR and DSC Assays

**Figure 2:**
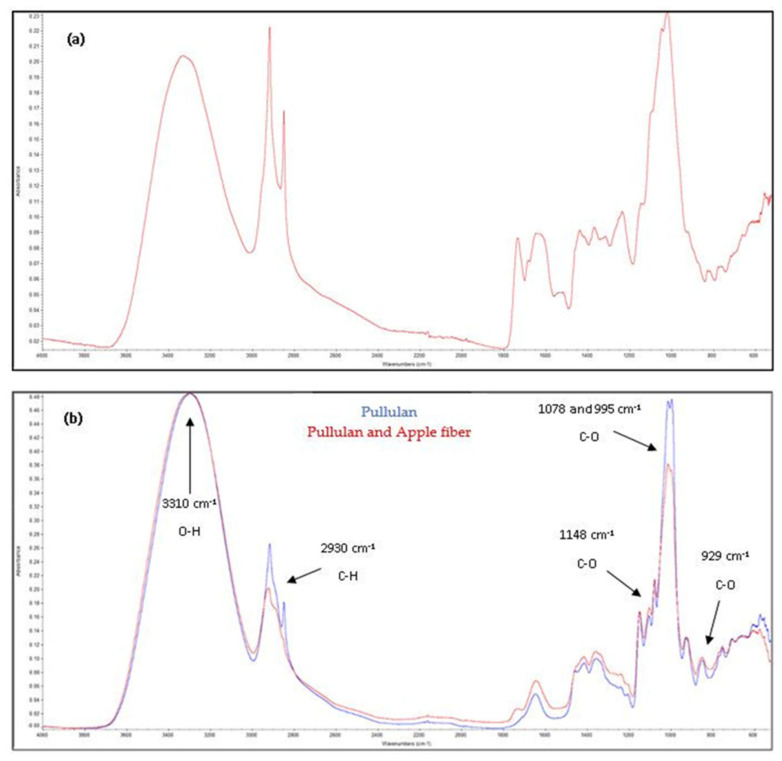
FTIR results of the raw apple fiber (**a**) and the biocomposite films (**b**).

Figure 2a shows the FTIR spectra of the apple fiber (maximum absorbance = 0.23), which present a similar profile to that of the biocomposite films (Figure 2b). Apples are particularly rich in pectin, a type of soluble fiber, also a polysaccharide-like pullulan.

The FTIR spectrum of the pullulan film (Figure 2b) shows the infrared bands corresponding to CH vibrations at 2930 cm^−1^ and the stretching vibrations of the OH groups of the polymer at 3310 cm^−1^. Furthermore, the CO vibrations of the glycosidic and etheric bounds of the pullulan molecule were detected at 929 cm^−1^, 995 cm^−1^, 1078 cm^−1^, and 1148 cm^−1^ (maximum absorbance = 0.48) [12,21].

The FTIR spectrum of the pullulan–apple fiber film (Figure 2b) showed slight differences in the zone of 1800 to 1200 cm^−1^; however, a considerable decrease in the absorbance was noticed at 2930 cm^−1^, 1078 cm^−1^, and 995 cm^−1^, indicating that the apple fiber was successfully incorporated into the pullulan matrix, suggesting its interaction with the pullulan molecules via hydrogen bonds, which also corroborates the visual aspect of the biocomposite films and their surface morphology, as described above.

**Figure 3:**
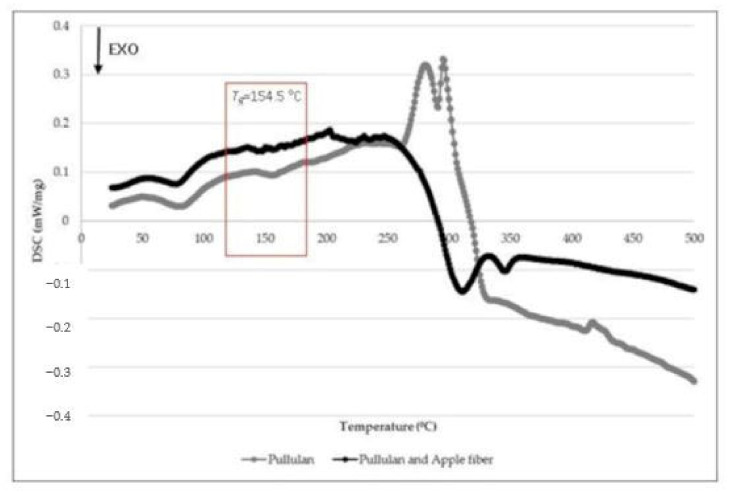
Differential Scanning Calorimetry (DSC) results of the biocomposite films.

The DSC results (Figure 3) revealed an increase in the glass transition temperature (*T_g_*) of the films when the apple fiber was incorporated into pullulan, the value of *T_g_* being 154.5 °C [9]. This *T_g_* increase suggests that the incorporation of apple fiber in the pullulan matrix changed the structure of the biocomposite films and, therefore, their mechanical properties [9]. To overcome this change in the mechanical properties, a higher percentage of glycerol (20%, *w*/*w*), used as a plasticizer, was added in the formulation of the biocomposite films, aiming to reduce the ratio of the crystalline to the amorphous region [22]. However, in previous work, we developed pullulan films incorporating rockrose essential oil, and only 15% (*w*/*w*) of glycerol was used, taking into account the potential plasticizer effect of the essential oil [12].

### 3.3. Grammage, Thickness, Mechanical, and Optical Properties

The grammage and thickness of the biocomposite films increased significantly (*p*-value < 0.05) with the apple fiber incorporation into the pullulan (Table 1). This increase was particularly substantial for the thickness, which, in the pullulan films, was 35.59 µm, and in the films with apple fiber was 155.87 µm. This result is a consequence of the rough surface of the upper side of the biocomposite films. It was previously described that plant-based fibers may present a certain degree of inconsistency [23], which also explains the roughness observed in the pullulan–apple fiber films.

The mechanical properties of the biocomposite films must be carefully evaluated, as they are important when considering the potential application of these films in food packaging [24]. In the present study, the Peak Elongation, the Tensile Index, the Tensile Strength, and the Elastic Modulus of the films were determined (Table 1). The Peak Elongation of the pullulan–apple fiber films was similar to the one of the pullulan films incorporated with rockrose essential oil (2.34%) previously developed [12], despite being significantly lower (*p*-value < 0.05) than the observed for the pullulan films (Table 1). The high value of Peak Elongation obtained for the pullulan films may be explained by the high percentage of glycerol used as a plasticizer. The high volumes of plasticizer decreased the cohesive forces amongst polymer chains, with the replacement of strong interactions with hydrogen bonds. Moreover, the hygroscopic behavior of glycerol enhanced the water absorption, which is also a film plasticizer. Consequently, the films became less rigid and more flexible [25]. It was also verified that the Tensile Index of the pullulan–apple fiber films was significantly higher (*p*-value < 0.05) when compared to the pullulan films (Table 1), indicating that the Tensile Strength of the pullulan–apple fiber films was also significantly higher (*p*-value < 0.05) (Table 1). Tensile Strength is the evaluation of the maximum strength of a film to withstand applied tensile stress [26]. Furthermore, the Elastic Modulus of the pullulan–apple fiber films was significantly higher (*p*-value < 0.05) than that of the pullulan films (Table 1).

Concerning the optical properties of the biocomposite films (Table 1), it was observed that when apple fiber was incorporated, there was a significant increase (*p*-value < 0.05) in color coordinates (L*, a*, and b*), which is consistent with their yellowish color. Moreover, a significant decrease (*p*-value < 0.05) in the transparency was verified in the pullulan–apple fiber films (Table 1), as it was also possible to visually observe.

### 3.4. Barrier Properties

The barrier properties of the biocomposite films were evaluated in terms of their permeability to water vapor and oil (Table 2) since good barrier properties are a steady-state indicator of biodegradable packaging materials [27].

Regarding the water vapor permeability, it was verified that no significant differences (*p*-value < 0.05) were observed in the WVTR when the apple fiber was incorporated into the biocomposite films (Table 2). However, a significant increase (*p*-value < 0.05) in the WVP was noticed (Table 2), which is related to the higher thickness of the pullulan–apple fiber films, as mentioned before. Previous works demonstrated that an increase in the thickness of films could result in an increase of WVP, since the resistance of the films to water vapor is decreased with the increase of the thickness. Therefore, a stagnant air layer is created, characterized by high partial pressure of water vapor on the inner film surface [14]. Similar results were observed for the OP of the biocomposite films developed (Table 2). It was assumed that glycerol, used as a plasticizer, increased the free volume between the pullulan molecules, which made oil permeate easily [14,27].

### 3.5. Contact Angles and Surface Free Energy

Surface wettability, which is frequently described by the water contact angle [28], was determined in the biocomposite films using a sessile drop method. Based on the Owens-Wendt approach, the surface free energy parameters of the films were calculated by the mean contact angles of water, diiodomethane, and ethylene glycol (Table 3). A significant increase (*p*-value < 0.05) of water contact angle in both sides of the films was verified when apple fiber was incorporated into the pullulan (Table 3), indicating an increase in the hydrophobicity of the developed biocomposite films (water contact angle is higher than 90° [29]), which is an important finding since food products often present high amounts of water. It is worth noting the hydrophobicity of the (rough) upper side of the pullulan–apple fiber films, presenting a water contact angle of 109.75°. It is well known that the roughness of the surfaces is directly related to the increase of the water contact angle, the so-called “lotus effect” [30], which we also previously explored [15]. Other authors reported that one of the most common design strategies for making an anti-wetting surface is to implement the surface roughness [31], which was clearly observed in the pullulan–apple fiber films now developed. While the intrinsic wettability of a material depends primarily on its surface free energy, the role of surface roughness becomes critical when fabricating anti-wetting surfaces [31].

It was observed that the total surface free energy (the sum of the polar and dispersive components of surface energy) of both types of films was not significantly different (*p*-value > 0.05) (Table 3) despite presenting significantly different (*p*-value < 0.05) surface free energy components (polar and dispersive) (Table 3), which will affect their interfacial interactions with liquids and thereby would show different wettability [31].

### 3.6. Antioxidant Activity

**Figure 4:**
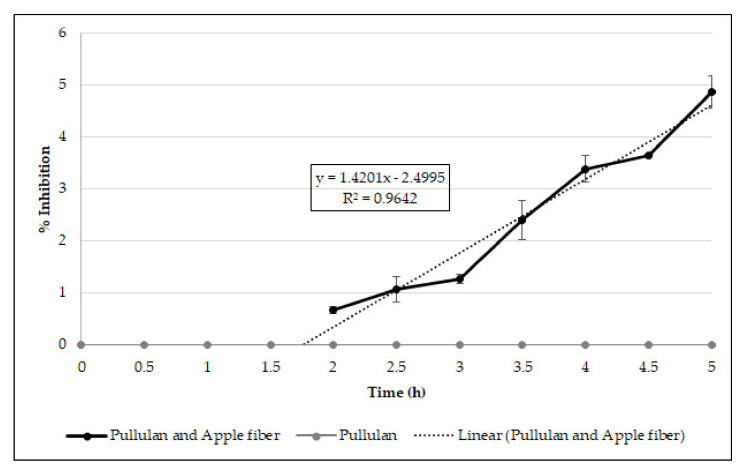
Antioxidant capacity of the biocomposite films measured by DPPH assay.

The antioxidant capacity of the biocomposite films was examined by quantifying the constant release of antioxidant compounds into the reaction mixture, evaluated by the DPPH free radical scavenging method, as shown in Figure 4.

The obtained results indicated that the DPPH scavenging capacity was clearly associated (R^2^ = 0.9642) with the time of reaction in the case of the pullulan–apple fiber films after the 2 h of reaction (before that, there was no antioxidant activity), while the pullulan films presented no capacity to scavenge the DPPH free radicals (Figure 4). These results may be explained by the presence of several polyphenolic compounds in apple fiber that were able to be released from the biocomposite films and to scavenge free radicals, as described previously by other authors [8,32,33].

Moreover, the pullulan–apple fiber films were able to hinder the lipid peroxidation, assessed by a β-carotene bleaching test (Table 4). The presence of antioxidants associated with apple fiber, like polyphenols, may be responsible for this protective effect against oxidation [8].

Overall, these findings evidence that not only can apple fiber be incorporated into pullulan films, reducing the quantity of pullulan needed to produce films, but also that when the apple fiber was added to the films, they acquired antioxidant properties, which could be beneficial to extending the shelf-life of packaged foods.

### 3.7. Antibacterial Properties

The antibacterial activity of the biocomposite films was evaluated by solid assay, with the inhibition zones diameters being measured (Table 5). It was observed that the pullulan–apple fiber films were able to cause bacterial cell retraction at the contact area with a clear inhibition zone for the tested Gram-positive and Gram-negative bacteria, contrariwise to what was noted for the pullulan films. Once more, the functionalization of the biocomposite films was achieved by the incorporation of apple fiber into the pullulan matrix, probably due to the bioactive compounds, like polyphenols, present in the apple fiber. Other authors developed polysaccharide-based edible coatings enriched with apple fiber and concluded that its incorporation into the formulations had a marked effect in reducing psychrophilic and mesophilic counts [8].

The results of antibacterial activity were also confirmed by optical microscopy. By observing the images presented in Table 6, it was possible to verify the microbial growth inhibition around the pullulan–apple fiber films for all the tested bacteria. Furthermore, the antibacterial effect was similar on both sides of the biocomposite films. Additionally, the images showed that the pullulan–apple fiber films maintain their integrity over the incubation period, contrary to what was observed for the pullulan films that dissolved along the incubation period. The obtained results suggest the relevance of the biocomposite films developed in this work for future applications in food packaging.

### 3.8. Biodegradability

A biodegradability test is a very valuable tool to help understand the environmental compatibility of developed materials. The soil biodegradability of food packaging materials was already stated. Particularly, the food packaging films produced with renewable biopolymers exhibited appealing soil biodegradable behaviors [34].

The biodegradability test of the developed biocomposite films was examined by a soil burial degradation test over 10 days (Table 4). It was verified that both types of films were completely degraded after 10 days, showing a weight loss of 100%. The addition of the apple fiber to the pullulan did not alter the biodegradability of the biocomposite films, making them appropriate for being put back in the environment without causing adverse effects, as other authors have also reported [35].

## 4. Conclusions

In this work, the incorporation of apple fiber in pullulan was shown to be a novel biopolymer basis to produce edible and bioactive films. The biocomposite films developed appeared to be resistant, flexible, and hydrophobic, together with their good visual aspect. Besides that, they were capable of scavenging free radicals, inhibiting lipid peroxidation, and inhibiting the growth of known foodborne pathogens.

The obtained results indicate that the pullulan–apple fiber biocomposite films developed in this work present a strong potential and are a promising material to develop new biodegradable alternatives to package foods, avoiding the use of traditional plastics.

## Figures and Tables

**Table 1:** Grammage, thickness, mechanical, and optical properties of the biocomposite films.

Properties ^1^		Pullulan	Pullulan and Apple Fiber	*p*-Value
Grammage (g/m^2^)		96.18 ± 4.63	131.52 ± 2.78	0.001 *
Thickness (µm)		35.59 ± 6.54	155.87 ± 11.35	<0.001 *
Mechanical	Peak Elongation (%)	221.51 ± 39.07	2.81 ± 0.13	0.010 *
	Tensile Index (N m/g)	0.47 ± 0.06	4.18 ± 0.45	0.004 *
	Tensile Strength (N/m)	42.05 ± 4.81	550.33 ± 57.37	0.004 *
	Elastic Modulus (MPa)	4.51 ± 0.70	286.39 ± 44.12	0.008 *
Optical	L* (lightness)	28.77 ± 2.08	39.30 ± 0.20	0.012 *
	a* (redness)	−0.12 ± 0.01	0.39 ± 0.07	0.020 *
	b* (yellowness)	−1.55 ± 0.14	5.93 ± 0.45	0.002 *
	Transparency (%)	94.13 ± 0.80	87.98 ± 0.24	0.003 *

^1^ Results shown as mean ± SD; * Significant result (*p*-value < 0.05).

**Table 2:** Barrier properties of the biocomposite films.

Permeability ^1^		Pullulan	Pullulan and Apple Fiber	*p*-Value
Water vapor	WVTR (g/m^2^ day)	55.50 ± 1.76	41.16 ± 7.06	0.065
	WVP (g/Pa day m)	1.49 × 10^-6^ ± 4.75 × 10^−8^	4.85 × 10^-6^ ± 8.32 × 10^−7^	0.020 *
Oil	OP (g mm/m^2^ day)	3.43 ± 0.08	124.21 ± 9.55	0.002 *

^1^ Results shown as mean ± SD; * Significant result (*p*-value < 0.05).

**Table 3:** Contact angles and surface free energy of the biocomposite films.

Properties ^1^	Pullulan		Pullulan and Apple Fiber		*p*-Value
	Lower Side ^a^	Upper Side ^b^	Lower Side (Smooth) ^c^	Upper Side (Rough) ^d^	
Water contact angle (°)	66.17 ± 1.79	64.99 ± 3.11	99.69 ± 2.11	109.75 ± 4.83	<0.001 ^ac^* <0.001 ^bd^*
Diiodomethane contact angle (°)	31.42 ± 1.32	37.05 ± 0.43	44.72 ± 1.87	41.82 ± 2.02	0.001 ^ac^* 0.049 ^bd^*
Ethylene glycol contact angle (°)	59.06 ± 1.95	49.93 ± 0.78	44.72 ± 1.87	41.82 ± 2.02	0.001 ^ac^* 0.011 ^bd^*
Total surface free energy, ɤT (mN/m)	40.92 ± 2.04	43.10 ± 2.15	42.43 ± 1.39	41.33 ± 0.52	0.361 ^ac^ 0.288 ^bd^
Polar component, ɤP (mN/m)	27.62 ± 1.37	31.25 ± 1.55	1.13 ± 0.33	2.95 ± 0.43	0.001 ^ac^* <0.001^bd^*
Dispersive component, ɤD (mN/m)	13.28 ± 0.65	11.84 ± 0.58	41.29 ± 1.37	38.38 ± 0.29	<0.001^ac^* <0.001 ^bd^*

^1^ Results shown as mean ± SD; superscript letters (a, b, c, d) identify the pairs of samples under statistical comparison; * Significant result (*p*-value < 0.05).

**Table 4:** Antioxidant activity (β-carotene bleaching test) and weight loss (biodegradability) of the biocomposite films.

Properties ^1^		Pullulan	Pullulan and Apple Fiber	*p*-Value
β-carotene bleaching test	Inhibition (%)	0.00 ± 0.00	0.96 ± 0.06	0.001 *
Biodegradability	Weight loss (%)	100.00 ± 0.00	100.00 ± 0.00	>0.05

^1^ Results shown as mean ± SD; * Significant result (*p*-value < 0.05).

**Table 5:** Antibacterial properties of the biocomposite films.

Diameters of Inhibition Zones ^1^	Pullulan	Pullulan and Apple Fiber		*p*-Value
		Lower Side (Smooth)	Upper Side (Rough)	
*Staphylococcus aureus* ATCC 25923	0.00 ± 0.00 (−)	6.00 ± 0.00 (+)	6.00 ± 0.00 (+)	<0.001 *
*Listeria monocytogenes* LMG 16779	0.00 ± 0.00 (−)	6.00 ± 0.00 (+)	6.00 ± 0.00 (+)	<0.001 *
*Enterococcus faecalis* ATCC 29212	0.00 ± 0.00 (−)	6.00 ± 0.00 (+)	6.00 ± 0.00 (+)	<0.001 *
*Bacillus cereus* ATCC 11778	0.00 ± 0.00 (−)	6.00 ± 0.00 (+)	6.00 ± 0.00 (+)	<0.001 *
*Salmonella typhimurium* ATCC 13311	0.00 ± 0.00 (−)	6.00 ± 0.00 (+)	6.00 ± 0.00 (+)	<0.001 *
*Escherichia coli* ATCC 25922	0.00 ± 0.00 (−)	6.00 ± 0.00 (+)	6.00 ± 0.00 (+)	<0.001 *
*Pseudomonas aeruginosa* ATCC 27853	0.00 ± 0.00 (−)	6.00 ± 0.00 (+)	6.00 ± 0.00 (+)	<0.001 *

^1^ Results shown as mean ± SD; (−) bacterial growth on the top of the films; (+) bacterial cell retraction at the contact area with a clear inhibition zone; * Significant result (*p*-value < 0.05).

**Table 6:** Optical microscopy images of antibacterial activity (Magnification: 400×).

Pullulan	Pullulan and Apple Fiber	
	Lower Side (Smooth)	Upper Side (Rough)
*Staphylococcus aureus* ATCC 25923		
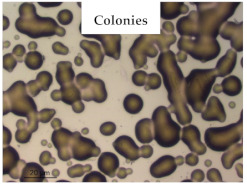	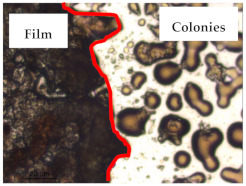	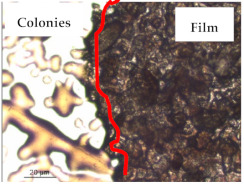
*Listeria monocytogenes* LMG 16779		
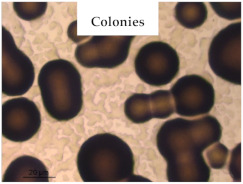	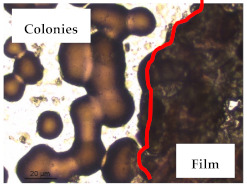	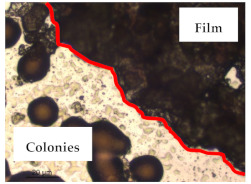
*Enterococcus faecalis* ATCC 29212		
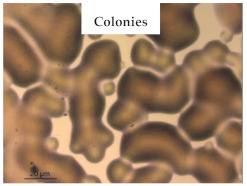	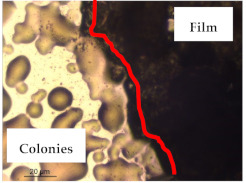	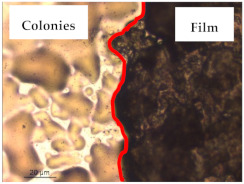
*Bacillus cereus* ATCC 11778		
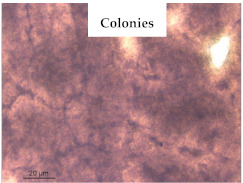	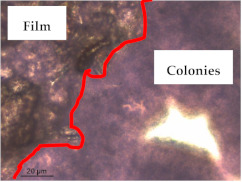	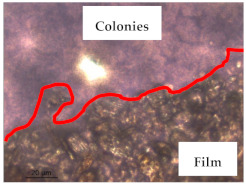
*Salmonella Typhimurium* ATCC 13311		
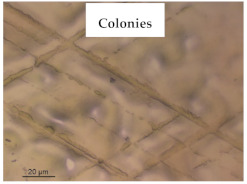	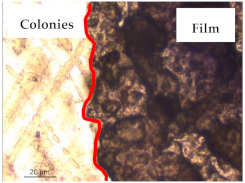	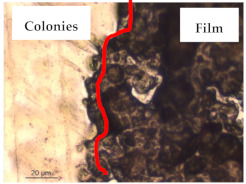
*Escherichia coli* ATCC 25922		
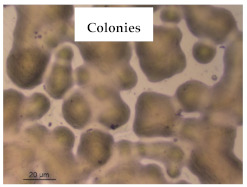	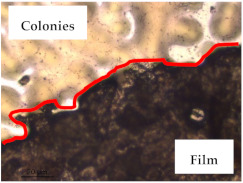	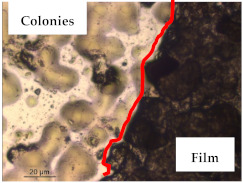
*Pseudomonas aeruginosa* ATCC 27853		
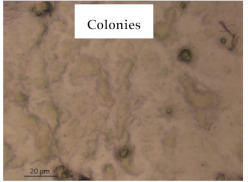	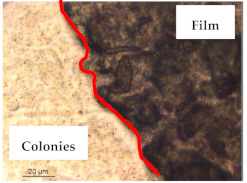	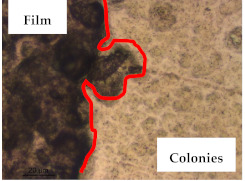

## Data Availability

The data presented in this study are available on request from the corresponding author.
